# The Potential of Intra-Articular Therapies in Managing Knee Osteoarthritis: A Systematic Review

**DOI:** 10.3390/clinpract14050157

**Published:** 2024-09-25

**Authors:** Ciprian Vasile Pojala, Sebastian Toma, Cristea Costache, Tunde Peter, Cristiana Elena Pojala, Nadinne Alexandra Roman, Lorena Dima

**Affiliations:** 1 Department of Fundamental, Preventive, and Clinical Disciplines, Faculty of Medicine, Transilvania University of Brasov, 500036 Brasov, Romaniacristea_costache@yahoo.com (C.C.); peter.tunde@gmail.com (T.P.);; 2 Department of Radiology and Medical Imaging, The University of Medicine and Pharmacy of Craiova, 200349 Craiova, Romania

**Keywords:** knee, osteoarthritis, hyaluronic acid, platelet-rich plasma

## Abstract

Background: Knee osteoarthritis (KOA) is a common degenerative and progressive joint disorder that negatively influences patients’ quality of life. Intra-articular therapies, such as hyaluronic acid (HA) and platelet-rich plasma (PRP), have garnered attention for their potential to manage osteoarthritis OA symptoms effectively. This systematic review aims to identify the effectiveness and safety of HA and PRP treatment modalities in treating KOA. Methods: A literature search was conducted across MEDLINE (PubMed), Web of Science Core Collection, and Science Direct Collection Elsevier. Twenty-three randomized controlled trials, cohort studies, and observational studies were included in the review. The selection criteria focused on studies published in English within the last 10 years, involving subjects with KOA treated with intra-articular injections of HA or PRP and reporting on pain, function, or overall treatment efficacy outcomes. Results: The analysis showed that both HA and PRP significantly improve functionality and reduce pain in KOA patients. High molecular weight HA consistently reduced pain and improved joint mobility in various studies. PRP had better long-term outcomes when combined with HA, leading to greater pain reduction and functional improvement. Both therapies had generally favorable safety profiles, with only minor adverse events reported. However, there were potential biases identified across the studies, such as selection, performance, detection, and reporting biases, which impacted the reliability of the results. Conclusions: Intra-articular treatments with HA and PRP show promise in managing knee osteoarthritis, with personalized treatment plans and further research needed to confirm these findings.

## 1. Introduction

Osteoarthritis (OA) is an increasingly prevalent condition in the general population, known for its disabling effects and significant burden on patients and healthcare systems [1,2,3].

It is a slowly progressive, non-inflammatory synovial joint disease, commonly called “wear and tear osteoarthritis” [4]. OA should be seen as a syndrome involving structural changes in the hyaline articular cartilage, subchondral bone, ligaments, capsule, synovium, and muscles. It typically affects joints that endure substantial stress, such as the hands, spine, knees, and hips [5]. The affected joint undergoes structural changes, including fibrillation, fissures, ulcerations, and complete loss of articular cartilage. This is accompanied by bone hypertrophy (osteophyte formation and subchondral bone sclerosis) and joint capsule thickening. These changes can be observed through radiological signs like reduced joint space, subchondral sclerosis, bone cysts, and osteophytes [6,7]. 

Globally, it is estimated that approximately 240 million people exhibit symptoms of OA, with an approximate percentage of 10 percent in men and 18% in women aged 60 years or older [8]. In an extensive study of people aged 60 and older 50-year-olds from England, approximately half of the subjects indicated that they had OA in at least one joint in the lower limb (weight-bearing joints) [9]. A recent study of individuals 20 years and older in Spain found that 29% had spine, hand, hip, and knee osteoarthritis [10]. Between 1997 and 2017, a study conducted in the UK using a comprehensive nationally representative database revealed 494,716 incident cases of clinical osteoarthritis (OA). This corresponds to a rate of 6.8 cases per 1000 people [11]. Furthermore, data from 1992 to 2013 demonstrated an increase in the prevalence of OA for subjects 45 years and older, from 29.2 to 40.5 [12].

Regarding KOA, the prevalence and incidence of this disease have been studied extensively compared to other lower limb joints [13]. New data suggests that the global prevalence of knee osteoarthritis at 16.0% and an incidence rate of 203 per 10,000 person-years. These findings offer valuable insights into the worldwide health burden of KOA. [14]. Another analysis carried out in China concluded that the prevalence of knee arthrosis is 14.6% [15]. Also, information regarding the Korean population suggests that the prevalence of radiographic OA of the knee in people aged 50 years or older was 35.1% [16].

The prevalence of typical radiographic knee arthrosis in patients aged 45 to 64 was reported at 17.6%, with the incidence of accelerated radiographic knee arthrosis at 3.7%. Additionally, the lifetime risk of developing symptomatic KOA is estimated to range from 14% to 45% [17,18].

In Spain, the incidence of hip arthrosis in individuals over 40 was reported at 5.1%, with a radiographic prevalence of 19.6% in those over 50, while symptomatic cases were observed at 4.2% [19,20]. For hand and ankle osteoarthritis, the prevalence in adults over 40 was 7.7% and 3.4%, respectively, and the estimated prevalence of hip osteoarthritis was higher in females (8.4%) than males (5.6%) [21,22,23]. 

According to recent data and projections for 2050 regarding OA, it is assumed that by 2050, there will be approximately 642 million people with KOA, 279 million people with hand OA, 62.6 million people with osteoarthritis of the hip, and 118 million people with other types of OA. These figures represent case increases from 2020 to 2050 of 74.9% for KOA, 48.6% for osteoarthritis of the hand, 78.6% for hip OA, and 95.1% (68.1–135.0) for other types of osteoarthritis [24].

The OA progression and development is influenced by an association of local and systemic factors, although its exact cause remains unclear [25,26].

The interplay of various factors is essential in the beginning of the condition and progression of OA. Key contributors include biomechanical changes related to aging, injuries, obesity, altered bone metabolism linked to metabolic syndrome, the influence of cytokines and related enzymes, as well as genetic predispositions [27,28].

Primary osteoarthritis results from cartilage breakdown and can affect any joint, particularly the fingers, spine, hips, and knees. Secondary osteoarthritis arises from damage to the articular cartilage due to injury, overuse, obesity, or joint instability [29,30].

Considering the diverse phenotypes of OA and its effects on multiple tissues, including cartilage, synovial tissue, bone, and even bone marrow, therapeutical strategies need to be customized to address each case’s specific characteristics.

Recently, there has been a continued focus on developing treatments aimed at halting or slowing the OA progression. Many of these treatments remain in clinical trials, and as our understanding of the condition’s pathophysiology advances, new therapies are anticipated [31,32,33].

Due to OA’s detrimental impact on quality of life, various therapies focus on relieving symptoms and improving patient well-being. However, no treatment has yet been found to delay or prevent OA or to provide long-term relief from joint damage and symptoms. Treatment strategies are typically tailored to the severity and duration of the patient’s symptoms, allowing for more personalized and effective care [34]. OA management typically involves lifestyle modifications, patient education on secondary prevention methods, physiotherapy, and pain management [35].

The 2019 “Osteoarthritis Research Society International” OARSI recommendations emphasize topical nonsteroidal anti-inflammatory drugs (NSAIDs) and exercise as key elements in a patient-centered approach [36]. Additionally, oral NSAIDs, intra-articular (IA) corticosteroids, and hyaluronan are strongly recommended for individuals with knee osteoarthritis, with treatment options tailored to the specific type of OA [36,37]. In addition, IA injections of hyaluronic acid (HA) have emerged as a non-surgical alternative for individuals who do not respond to first-line pharmacological treatments, lack a surgical indication, or prefer to avoid surgery [38,39].

Platelet-rich plasma (PRP) injections are another non-surgical treatment option for patients [40]. A study by Lin et al. [41] concluded that intra-articular treatment with leukocyte-poor PRP may produce clinical amelioration for up to one year in subjects with mild to moderate KOA. The numerous anabolic growth factors found in PRP, such as FGF, TGF-β1, TGF-β2, and EGF, along with anti-inflammatory cytokines like IL-1ra, sTNF-R1, sTNFRII, IL-4, IL-10, IL-13, and IFNγ, may contribute to modifying the pathological process of knee osteoarthritis [42].

Mesenchymal stem cell (MSC) treatment is another non-surgical modality. The HA efficiency and mesenchymal stem cells have been compared in several studies. However, several aspects remain under discussion, including the optimal cell source, their characteristics, and the appropriate dosage. Additional research is required to address these ongoing uncertainties [43]. 

In this systematic review, we aim to critically evaluate the potential of intra-articular therapies in managing knee osteoarthritis. The primary reference endpoints in the manuscript were pain and functionality. These were assessed using widely recognized scales and indices, such as the “Visual Analog Scale” (VAS) [44] for pain and the “Western Ontario and McMaster Universities Arthritis Index” (WOMAC) [45] for functionality. In addition to pain and functionality, other endpoints were also considered, including radiological outcomes, Quality of Life, safety, and adverse effects. 

## 2. Materials and Methods

### 2.1. Search Strategy

To evaluate the efficacy of intra-articular HA viscosupplementation and other therapies for KOA, a comprehensive literature search was conducted across three major databases: MEDLINE (PubMed), Web of Science Core Collection, and Science Direct Collection Elsevier. The search was performed from 4–8 March 2024.

### 2.2. Search Terms and Inclusion Criteria

The search was performed by using the association of targeted keywords to ensure a comprehensive and focused approach to gathering relevant information. Medical Subject Headings (MeSH) terms were used. The leading search terminology used: “Knee”, “Osteoarthritis”, “Hyaluronic acid”, “Viscosupplementation”, and “Intra-articular injection”. Supplementary information regarding the search strategy can be found in Supplementary Materials “Detailed Search Strategy”.

These terms were combined using Boolean operators (AND, OR) to refine the search results. Only papers written in English were included.

The inclusion criteria for the studies were:

1. Studies involving patients diagnosed with knee osteoarthritis.
2. Studies that evaluated using hyaluronic acid or PRP for intra-articular injections.
3. Studies reported on pain, function, or overall treatment efficacy outcomes.
4. Both randomized controlled trials (RCTs) and observational studies were included to capture a wide range of evidence.
5. Papers published in the last 10 years.

### 2.3. Screening Process

1. Duplicate Removal: All retrieved articles were imported into a reference management software (Zotero 6.0.36) where duplicates were identified and removed (C.V.P.).
2. Title and Abstract Screening: The first step in the screening process was evaluating articles’ titles and abstracts. Studies that did not meet the inclusion criteria were excluded at this stage. C.V.P. and C.E.P., under the supervision of L.D., conducted the initial screening of titles and abstracts. They reviewed the abstracts to assess the relevance of the studies according to the predefined inclusion criteria.
3. Full-Text Review: The full texts of articles that passed the initial screening were obtained and reviewed in detail. This step ensured that all included studies met the predefined criteria and provided sufficient data for analysis. Full-text articles were obtained for all studies that passed the abstract screening stage. C.V.P., C.E.P., and N.A.R. were responsible for the detailed full-text review, examining each article for alignment with the inclusion criteria, including study design, participant characteristics, interventions, outcomes, and the quality of the study. Key aspects such as sample size, duration of follow-up, types of interventions, and outcome measures were carefully evaluated.

C.V.P. and C.E.P. assisted in the review process, mainly focusing on clinical relevance and the applicability of findings.

4. Consensus and Validation: L.D. and S.T. validated the final list of included studies, ensuring the selection was rigorous and aligned with the review’s objectives. Any discrepancies or uncertainties during the full-text review were resolved through group discussion, with N.A.R. ensuring methodological soundness.

C.E.P. and C.C. formally analyzed the bias assessments, identifying potential impacts on the study outcomes and overall reliability.

N.A.R. and L.D. worked together to interpret the results of the bias analysis. 

### 2.4. Data Extraction and Analysis

For each study included in the review, the following data were extracted:

- Study design (e.g., RCT, cohort study, observational study);
- Sample size;
- Participant demographics (age, gender distribution);
- Duration of follow-up;
- Types of interventions (specific HA formulations, combinations with other treatments);
- Outcome measures (e.g., VAS for pain, WOMAC for function);
- Key findings and results.

The exclusion criteria encompassed:

Non-Original Research: Studies that are review articles, commentaries, letters to the editor, or editorial pieces.

Animal Studies: Research that involves non-human subjects or in vitro experiments.

Unpublished Data: Abstracts, conference proceedings, dissertations, and theses that are not peer-reviewed full-text articles.

Incomplete Data: Studies lacking detailed methodology or results, making it impossible to assess the quality and outcomes of the intervention.

Irrelevant Interventions: Research focusing on treatments other than intra-articular HA or PRP, such as oral medications, physical therapy, or surgical interventions.

Short-Term Follow-Up: Follow-up research lasting less than three months, which may lack consistent information regarding efficiency and the interventions’ safety.

Small Sample Size: Studies with fewer than 20 participants per treatment group may not provide sufficient statistical power to draw reliable conclusions [46].

Duplicate Publications: Multiple publications of the same study, where the most comprehensive version will be included.

Outdated Research: Studies published before 2015 to ensure the inclusion of more recent data and methodologies.

Inadequate Outcome Measures: Research that does not use validated outcome measures such as Visual Analog Scale (VAS), The Western Ontario and McMaster Universities Arthritis Index (WOMAC), Knee Injury and Osteoarthritis Outcomes Score (KOOS), or similar standardized tools to assess pain and function.

- Selection Bias

Selection bias happens when the individuals chosen to participate in a study do not accurately represent the wider population, which can lead to skewed or inaccurate results. This issue can arise due to various factors such as the method of participant selection, non-response bias, or exclusion criteria, and it is important to address and minimize selection bias in research studies to ensure the validity of the findings [47]. This bias can affect the generalizability of the study results. The bias analysis was performed using the Cochrane Collaboration risk of bias tool.

- Performance Bias

Performance bias refers to a situation where there is uneven treatment provided to participants in different groups, unrelated to the actual intervention under study. This imbalance typically occurs because blinding is not implemented effectively [48].

- Detection Bias

Detection bias arises when the method of assessing outcomes differs between groups, often due to inadequate blinding of outcome assessors [49].

**Figure 1:**
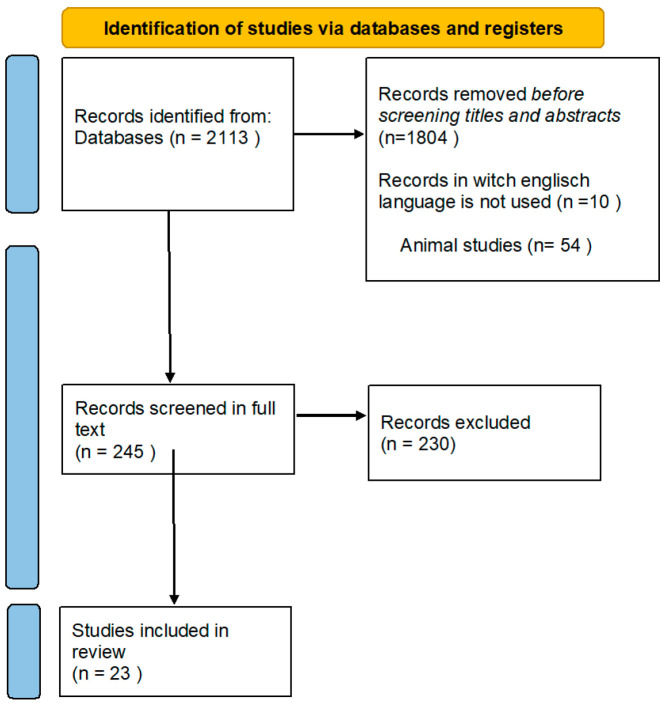
Diagram flow of the search strategy and study selection.

The extracted data were subsequently synthesized to offer a comprehensive overview of the current evidence on HA viscosupplementation’s effectiveness in treating KOA. The refinement process results are shown in Figure 1. The study selection was performed according to PRISMA guidelines [50].

## 3. Results

In Table 1 are expanded the informations regarding the analysed apaers, including the type of the study, the number of participants and groups, intervention types and main outcomes.

### 3.1. Characteristics of the Analyzed Studies

#### Participants

The number of participants in these studies varies widely, with the smallest study including 44 participants (Babu et al. [51]) and the largest encompassing 284 participants (Blicharski et al. [53]). The duration of the studies ranges from a few months to several years, with the shortest study lasting 3 months (Raeissadat et al. [52]) and the longest spanning 5 years (Galluccio et al. [61]). Gender distribution varies, with some studies having a higher proportion of female participants, such as Babu et al. [51] with 73% women, while others like Blicharski et al. [53] include a more balanced mix of 105 males and 179 females. The age range of participants also varies, with the youngest mean age being around 36 years (Babu et al. [51]) and the oldest mean age around 66 years (Perruchet et al. [60]).

### 3.2. Hyaluronic Acid (HA) Studies

Types of Studies: The studies focusing on Hyaluronic Acid (HA) included a variety of study designs, with randomized controlled trials (RCTs) being the most common due to their ability to minimize bias. Examples include research performed by Raeissadat et al. [52] and Blicharski et al. [53]. These RCTs were complemented by prospective observational studies, conducted by Acharya et al. [55] and Hill et al. [56], which tracked outcomes over time without intervention from researchers. Cohort studies, like the long-term evaluation by Galluccio et al. [61], followed a group of individuals who received specific HA treatments to observe effects over extended periods.

Participants: The number of participants in HA studies varied widely. The smallest study included 44 participants (Babu et al. [51]), while the largest encompassed 284 participants (Blicharski et al. [53]). The duration of these studies ranged from a few months to several years, with the shortest study lasting 3 months (Raeissadat et al. [52]) and the longest spanning 5 years (Galluccio et al. [61]). Gender distribution also varied, with some studies having a higher proportion of female participants (e.g., Babu et al. [51]), while others included a more balanced mix (e.g., Blicharski et al. [53]). The age range of participants also varied, with the youngest mean age around 36 years (Babu et al. [51]) and the oldest around 66 years (Perruchet et al. [60]).

Types of Interventions: The interventions in these studies involved different formulations of HA. For instance, Babu et al. used high molecular weight intra-articular HA (HMW-IAHA), Blicharski et al. [53] examined Hyruan ONE and Durolane, and Acharya et al. [55] evaluated Hylan G-F 20. Some studies, like that of Galluccio et al. [61], examined the effects of quarterly injections of Hyalubrix over five years. Additionally, some studies explored combining HA with other treatments, such as PRGF and ozone, as seen in Raeissadat et al.’s research [52].

Assessment Methods: Various scales were used to measure outcomes in HA studies. The VAS was commonly employed for pain evaluation, as seen in research papers by Babu et al. [51] and Raeissadat et al. [52]. The WOMAC was widely used to assess pain, stiffness, and physical function, featured in studies by Acharya et al. [55] and Nouri et al. [57]. The Kellgren–Lawrence Score, which assesses the radiological severity of osteoarthritis, was used in studies by Blicharski et al. [53].

Main Results: HA studies consistently showed significant improvements in pain and function. Babu et al. [51] reported a decrease in VAS scores from 8.53 to 5.97, and Raeissadat et al. [52] found that HA treatments led to substantial pain reduction. Functional improvements were also significant, as indicated by reductions in WOMAC scores across multiple studies. Long-term efficacy was highlighted in studies like Galluccio et al., where sustained pain relief and improved joint function were observed over five years with quarterly HA injections. The safety profile of HA was generally favorable, with minor side events reported, such as injection site pain and transient swelling, but no serious adverse events.

Bias Analysis: Regarding selection bias, studies with robust random sequence generation and allocation concealment, like Hill et al. [56], exhibited low bias. However, some research, like Yılmaz et al. [63] and Calvet et al. [54], lacked explicit details on random sequence generation, leading to unclear selection bias. Performance bias was minimized in studies like Blicharski et al. [53], which maintained blinding by having participants wear eye masks during injections. Conversely, some studies did not adequately implement blinding, resulting in high-performance bias. Detection bias was reduced in studies with blinded assessors, such as Raeissadat et al. [52], while others, like Galluccio et al. [61], had unclear detection bias due to a lack of information on blinding.

### 3.3. Platelet-Rich Plasma (PRP) Studies

Types of Studies: The studies focusing on platelet-rich plasma (PRP) included a range of study designs, with randomized controlled trials (RCTs) being the primary type due to their rigorous methodology. For example, Nouri et al. [57] conducted an RCT comparing HA, PRP, and their combination. Prospective observational studies, such as those conducted by Hill et al. [56], provided additional insights by tracking patient outcomes over time. These studies were instrumental in evaluating the long-term effects of PRP treatments.

Participants: The participant demographics in PRP studies also varied widely. Nouri et al.’s study [57] included 92 participants, while Hill et al.’s study [56] involved 93 participants. The age range of participants in PRP studies was broad, with some studies focusing on middle-aged adults and others including older populations. Gender distribution was also varied, with a mix of male and female participants across different studies.

Types of Interventions: PRP interventions in these studies often involved multiple injections. For instance, Nouri et al. [57] compared the effects of PRP alone, HA alone, and a combination of both. Raeissadat et al. [52] examined PRP with PRGF and found significant differences in pain and function compared to HA and ozone treatments. The specific formulations and protocols for PRP varied, with some studies focusing on leukocyte-rich PRP while others used leukocyte-poor PRP.

Assessment Methods: The studies used similar assessment scales to HA studies, with the VAS and WOMAC being the most common. Nouri et al. [57] used these scales to evaluate pain and function, finding significant improvements in both measures after PRP treatment. The Lequesne index was also employed in some studies to assess the negative impact of KOA and the efficiency of the interventions.

Main Results: PRP studies consistently depicted major improvements in pain and function/functionality, often surpassing the results seen with HA treatments. Hence, Raeissadat et al. [52] found that PRP and PRGF groups had meaningfully lower VAS and WOMAC scores at 12 months than the HA group. Nouri et al. [57] showed that combining PRP with HA led to superior outcomes compared to either treatment alone, with significant reductions in WOMAC, VAS, and Lequesne scores at 2 and 6 months post-treatment. The safety profile of platelet-rich plasma (PRP) was found to be favorable overall, with the majority of studies reporting only minor adverse events such as injection site pain and transient swelling, which were similar to those noted in hyaluronic acid (HA) studies.

Bias Analysis: The risk of bias in PRP studies was generally well managed. Selection bias was low in studies with robust randomization, such as those by Nouri et al. [57]. Performance bias was minimized in studies that implemented effective blinding, like those by Raeissadat et al. [52]. However, some studies faced challenges with detection bias due to the use of subjective outcome measures like VAS and WOMAC, which are susceptible to participant reporting biases. Overall, the bias analysis indicated that while PRP studies were methodologically sound, there were areas where bias could potentially influence the outcomes, particularly in studies with less rigorous blinding protocols.

### 3.4. General Analysis of Biases

- Selection Bias

**Figure 2:**
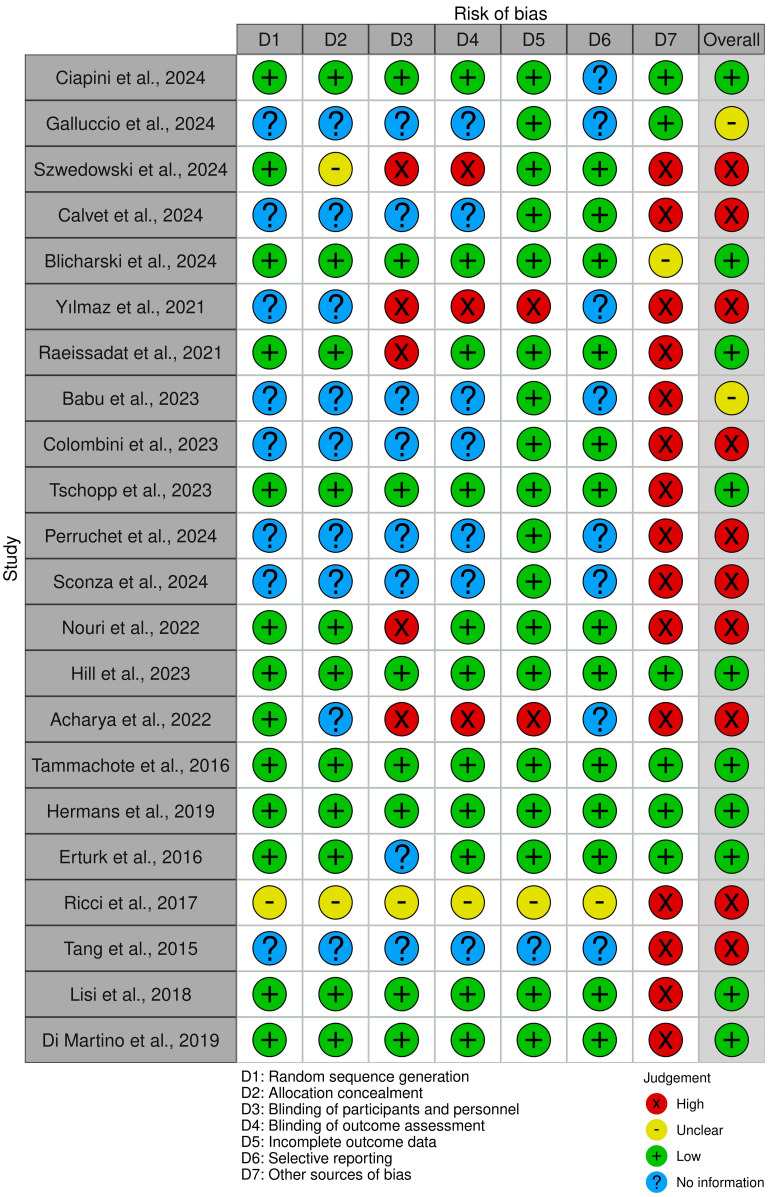
Quality analysis of the revised studies [51,52,53,54,55,56,57,58,59,60,61,62,63,64,65,66,67,69,70,71,72,73].

Studies with robust random sequence generation and allocation concealment methods exhibit low selection bias. The main analysis regarding biases in the investigated papers are depicted in Figure 2. For instance, Hill et al. [56] utilized a randomization.

Program of the SAS system, ensuring unbiased participant assignment and effectively minimizing selection bias. Similarly, Tammachote et al. [66] ensured allocation concealment by using computer-generated random numbers and sealed opaque envelopes, reducing the risk of selection bias. On the other hand, studies like Yılmaz et al. [63] and Calvet et al. [54] lacked explicit details on random sequence generation and allocation concealment, resulting in unclear or potentially high selection bias.

Many studies included in the analysis used specific inclusion criteria, which could lead to selection bias. For instance, studies by Raeissadat et al. [52] and Babu et al. [51] selected participants based on stringent criteria, potentially excluding those with more severe conditions or comorbidities common in the general population of osteoarthritis patients.

Several studies had a disproportionate number of female participants, such as Babu et al. [51], where 73% of participants were women. This imbalance could skew results if the intervention effects differ between genders.

- Performance Bias

Studies that successfully implement blinding of participants and personnel minimize this bias. For example, Blicharski et al. [53] maintained blinding by having participants wear eye masks during injections and ensured that other personnel were also blinded, leading to low-performance bias. Conversely, five studies [52,55,57,58,63] did not adequately implement blinding of participants and personnel, resulting in high-performance bias due to the potential influence of knowledge of treatment allocation on the outcomes.

Studies like Eturk et al. [69], which did not include a placebo group or used an active control, could be subject to performance bias due to participants’ and clinicians’ expectations influencing the outcomes.

- Detection Bias

Detection bias arises when the method of assessing outcomes differs between groups, often due to inadequate blinding of outcome assessors. Studies like those by Lisi et al. [72] and Di Martino et al. [73], which included blinded assessors, help reduce this bias. Effective blinding of those assessing outcomes reduces this risk. Raeissadat et al. [52] exemplified low detection bias by ensuring that outcome assessments were conducted by blinded evaluators who were unaware of the treatment allocation. In contrast, studies like Galluccio et al. [61] and Calvet et al. [54] did not provide clear information on whether blinding of outcome assessment was implemented, leading to unclear or potentially high detection bias.

Using subjective outcome measures such as pain scales (VAS) and self-reported function scales (WOMAC) can introduce detection bias, as these measures are susceptible to participant reporting biases.

### 3.5. Radiological Outcomes

Radiological outcomes were assessed in several studies included in this review, primarily using the Kellgren–Lawrence (KL) grading system to evaluate the severity of osteoarthritis. For instance, Blicharski et al. [53] reported significant improvements in KL scores following the administration of cross-linked sodium hyaluronate, indicating a reduction in the progression of structural joint damage. Similarly, Galluccio et al. [61] noted that long-term quarterly injections of Hyalubrix were associated with stable or improved KL scores over five years, suggesting a protective effect against further joint degeneration. These findings highlight the potential of intra-articular hyaluronic acid (HA) treatments not only to alleviate symptoms but also to slow the structural progression of knee osteoarthritis.

### 3.6. Quality of Life

Quality of life (QoL) was a critical endpoint evaluated in multiple studies through tools such as the Short Form Health Survey (SF-36). After HA injections, Acharya et al. [55] observed significant enhancements in QoL scores, particularly in physical functioning and general health domains. These improvements were consistent across different age groups, although older patients demonstrated slightly less pronounced gains than younger participants. Hill et al. [56] also found that patients receiving FlexPro MD^®^ experienced significantly improved SF-36 scores, positively impacting overall well-being, pain relief, and functional enhancement. These results underscore the holistic benefits of intra-articular therapies, extending beyond joint-specific outcomes to broader aspects of patient health and daily living.

### 3.7. Safety and Adverse Events

The safety profile of intra-articular HA and PRP treatments was generally favorable across the reviewed studies, with most reporting only minor adverse events. Raeissadat et al. [52] documented mild and transient injection site pain and swelling as the most common adverse effects following PRP and HA injections, with no significant long-term complications. Similarly, Galluccio et al. [61] confirmed the long-term safety of HA, noting the absence of severe adverse events over a five-year follow-up period. These findings suggest that both HA and PRP are well-tolerated by patients, with a low incidence of adverse effects, supporting their use as safe treatment options for knee osteoarthritis.

## 4. Discussion

The systematic review of 23 studies on various interventions for knee osteoarthritis (KOA) reveals insightful findings regarding their efficacy, safety, and practical application. The review highlights diverse research designs, including prospective cohort studies, randomized controlled trials (RCTs), and observational studies, each contributing unique insights into KOA treatment.

The management of KOA is a complex and significant challenge in clinical practice. It necessitates a multifaceted approach that includes both pharmacological and non-pharmacological interventions. Intra-articular therapies have garnered considerable attention due to their potential to identify localized relief with reduced systemic side effects. This discussion synthesizes findings from various studies to evaluate the efficacy, safety, and potential biases of intra-articular therapies, particularly hyaluronic acid (HA) and platelet-rich plasma (PRP), in treating knee OA.

### 4.1. Efficacy of Hyaluronic Acid (HA)

Hyaluronic acid (HA) naturally exists in the synovial fluid of joints, where it acts as both a lubricant and shock absorber. Injections of HA within the joint aim to restore the viscoelastic properties of the synovial fluid, leading to reduced pain and improved joint function. The efficacy of HA in knee OA has been well-documented across multiple studies. Babu et al. [51] reported significant ameliorations in pain and functional outcomes following HA injections, with VAS scores decreasing from 8.53 to 5.97 over a 90-day period. This improvement in pain was complemented by enhancements in KOOS scores, indicating overall and specific improvements in joint function. Similarly, Blicharski et al. [53] found that treatment with cross-linked sodium hyaluronate effectively managed mild to moderate knee OA, as evidenced by significant improvements in WOMAC and Likert Scale scores.

Long-term studies further support the efficacy of HA. Galluccio et al. [61] conducted a five-year cohort study using quarterly intra-articular injections of Hyalubrix, demonstrating sustained pain relief and improved joint function over the study period. This study’s findings are crucial as they indicate the potential for HA to provide long-term benefits with minimal adverse effects, supporting its use as a sustainable treatment option for knee OA. HA injections are effective, but their efficacy can vary based on the molecular weight and formulation of the HA used. Studies like those by Acharya et al. [55], which used Hylan G-F 20, also support the positive outcomes associated with HA, further emphasizing the role of specific formulations in achieving optimal results.

HA seems to be ad effective treatment for knee osteoarthritis (KOA), renowned for significantly enhancing the viscoelastic properties of synovial fluid. This improvement plays a crucial role in joint lubrication and shock absorption. Numerous studies have investigated the efficacy of HA injections, each offering valuable insights into its therapeutic potential.

Efficacy of HA in Pain Reduction: The studies reviewed consistently demonstrate that HA injections significantly reduce pain among patients with KOA. For instance, Babu et al. (2023) [51] reported a notable decrease in Visual Analog Scale (VAS) scores from 8.53 at baseline to 5.97 at 90 days post-injection, indicating substantial pain relief. This aligns with findings from Blicharski et al. (2023) [53], who observed significant improvements in pain, as measured by both the VAS and the Western Ontario and McMaster Universities Osteoarthritis Index (WOMAC) scores, following treatment with cross-linked sodium hyaluronate. The reduction in pain is particularly noteworthy given the chronic and often debilitating nature of KOA pain, underscoring HA’s role as a viable non-surgical intervention.

Functional Improvements and Quality of Life: Beyond pain relief, HA injections have also been associated with improved joint function and overall quality of life. Acharya et al. (2022) [55] reported significant enhancements in physical function, as evidenced by reductions in WOMAC scores and improvements in the Short Form (SF)-36 Health Survey results. Similarly, Galluccio et al. (2022) [61] conducted a five-year cohort study demonstrating that quarterly intra-articular HA injections reduced pain and sustained joint function improvements over the long term. This finding is critical as it suggests that HA injections can provide durable benefits, maintaining joint function and QoL in patients with KOA.

Long-Term Efficacy and Safety: The long-term efficacy of HA was further supported by studies like Galluccio et al. (2022) [61], which showed that consistent quarterly injections over five years resulted in sustained pain relief and functional improvements without significant adverse effects. This long-term benefit is corroborated by studies like Blicharski et al. (2023) [53], which highlighted that cross-linked HA formulations provide effective symptom relief with minimal side effects. The safety profile of hyaluronic acid (HA) is overwhelmingly favorable, with the majority of studies reporting only minor and transient adverse events, such as injection site pain and mild swelling. This solidifies HA as a secure option for long-term management of KOA, particularly for patients who are either unsuitable for surgery or prefer non-surgical treatments.

Variability in Outcomes: It’s important to recognize that the effectiveness of HA injections can differ based on various factors, including the molecular weight of the HA formulation, the frequency of injections, and patient characteristics such as age, BMI, and the severity of osteoarthritis. For example, Acharya et al. (2022) [55] found that while all age groups experienced pain relief, older patients showed slightly less improvement in function compared to younger patients. Additionally, studies like those by Babu et al. (2023) [51] and Blicharski et al. (2023) [53] emphasized that higher molecular weight HA formulations tend to provide more substantial and prolonged benefits compared to lower molecular weight formulations.

### 4.2. Efficacy of PRP

PRP is an autologous blood product containing a high concentration of platelets, growth factors, and cytokines. These components are believed to promote tissue repair and modulate inflammation. PRP injections have become popular as a treatment for knee osteoarthritis (OA) because of their potential regenerative properties [74]. Several robust studies have unequivocally demonstrated the efficacy of platelet-rich plasma (PRP) in reducing pain and improving joint function, consistently outperforming Hyaluronic Acid (HA) in providing long-term benefits. In a randomized clinical trial conducted by Raeissadat et al. [52], the findings strongly support the assertion that PRP and Platelet-Rich Growth Factor (PRGF) groups exhibit significantly lower VAS and WOMAC scores at 12 months compared to the HA group, conclusively indicating that PRP may indeed offer more sustained relief from symptoms of osteoarthritis.

When combined with HA, the synergistic potential of PRP is also noteworthy. Nouri et al. [57] explored the effects of combining PRP with HA and found that this combination led to superior pain reduction and functional improvement compared to either treatment alone. This study demonstrated significant differences in WOMAC, VAS, and Lequesne scores at 2 and 6 months post-treatment, indicating that combining PRP with HA can enhance the therapeutic effects and potentially prolong the duration of symptom relief.

Di Martino et al. [73] conducted a comprehensive five-year follow-up study comparing platelet-rich plasma (PRP) and hyaluronic acid (HA). The study unequivocally demonstrated that both treatments effectively improved knee functional status and symptoms over time. Despite the PRP group showing higher values in the International Knee Documentation Committee (IKDC) subjective score at the final evaluation, the study found that PRP’s overall clinical improvement was not significantly superior to HA. This finding suggests that while PRP may offer particular advantages, particularly in long-term maintenance of joint function, HA remains a viable and effective option for many patients.

The superiority of PRP in Long-Term Outcomes: The studies reviewed suggest that PRP may offer superior long-term benefits compared to HA, particularly in pain reduction and functional improvement. Raeissadat et al. (2021) [52] conducted a randomized clinical trial comparing PRP, Plasma Rich in Growth Factors (PRGF), HA, and ozone therapy. They found that the PRP and PRGF groups demonstrated significantly lower VAS and WOMAC scores at 12 months post-treatment compared to the HA group. This indicates that PRP may provide more sustained symptom relief than HA, making it a promising alternative or adjunctive therapy for KOA.

Combining PRP and HA: The potential for synergistic effects when combining PRP with HA is another key finding from the reviewed studies. Nouri et al. (2020) [57] explored the combined use of PRP and HA, revealing that this combination led to superior pain reduction and functional improvement compared to either treatment alone. The study reported significant improvements in WOMAC, VAS, and Lequesne scores at 2 and 6 months post-treatment, suggesting that the combination of PRP and HA may enhance the therapeutic effects and prolong the duration of symptom relief. This combination therapy could represent an optimal strategy for patients who have not responded adequately to HA alone or who require more aggressive treatment to manage their symptoms.

Regenerative Potential and Mechanisms of Action: PRP’s regenerative potential is attributed to its high concentration of growth factors, such as transforming growth factor-beta (TGF-β), platelet-derived growth factor (PDGF), and vascular endothelial growth factor (VEGF). These factors are believed to be crucial in tissue healing and modulation of inflammation. The studies reviewed, such as those by Raeissadat et al. (2021) [52] and Di Martino et al. (2019) [73], underscore PRP’s ability to not only reduce pain but also promote cartilage repair and improve joint function over the long term. Di Martino et al. (2019) [73] conducted a five-year follow-up study comparing PRP and HA, concluding that while both treatments were effective, PRP showed higher International Knee Documentation Committee (IKDC) subjective scores at final evaluation, suggesting better maintenance of joint function over time.

Safety Profile and Adverse Events: The safety profile of PRP is generally favorable, with most studies reporting only minor and transient adverse events, such as injection site pain, transient swelling, and mild inflammatory reactions. Raeissadat et al. (2021) [52] noted that PRP and PRGF treatments were well-tolerated, with no significant safety concerns. However, it is essential to consider that PRP’s efficacy and safety can vary depending on the preparation method, platelet concentration, and individual patient factors. For example, the leukocyte-rich versus leukocyte-poor PRP debate is ongoing, with some studies suggesting that leukocyte-poor PRP may reduce inflammation more effectively, thereby enhancing patient outcomes.

Variability in Patient Response: Similar to HA, the response to PRP therapy can vary among patients, depending on factors such as the severity of osteoarthritis, the patient’s overall health, and the specific PRP preparation used. Di Martino et al. (2019) [73] highlighted that while PRP was effective in most patients, the improvement in symptoms and function varied, particularly among those with more advanced diseases. This variability underscores the need for personalized treatment approaches, where the choice between PRP, HA, or their combination is tailored to the patient’s needs and disease characteristics.

### 4.3. Comparative Efficacy and Safety

Comparative studies between HA and PRP indicate that both treatments are effective, but PRP might provide more durable benefits for some patients. For example, in studies where both treatments were evaluated, such as those by Di Martino et al. [73] and Nouri et al. [57], PRP often showed higher long-term efficacy in reducing pain and improving function. Safety profiles for both HA and PRP are generally favorable, with most studies reporting only minor adverse events like injection site pain and transient swelling. For instance, Raeissadat et al. [52] noted that PRP and PRGF treatments were well-tolerated, with no significant safety concerns, while Galluccio et al. [61] confirmed the safety of long-term HA use.

### 4.4. Potential Biases

Despite the promising findings, several potential biases must be considered. Selection bias is evident in many studies due to specific inclusion criteria, potentially excluding patients with severe comorbidities. Performance bias may arise from inadequate blinding, which could influence outcomes due to participants’ and clinicians’ expectations. Detection bias is also a concern, given the reliance on subjective measures like VAS and WOMAC. Long-term studies face attrition bias, and the handling of missing data varies across studies, potentially impacting results. Reporting bias, driven by selective outcome reporting, further complicates the interpretation of findings.

### 4.5. Implications for Clinical Practice and Future Research

The evidence supports intra-articular HA and PRP use in managing knee OA, with both therapies demonstrating significant improvements in pain and function. However, the variability in response highlights the need for personalized treatment plans. Combining HA and PRP may offer enhanced benefits, suggesting a potential avenue for optimizing therapeutic strategies. Future research should focus on large-scale, multicenter RCTs with robust blinding and long-term follow-ups to confirm these findings. Addressing biases through rigorous study design and comprehensive reporting will enhance the reliability of results. Moreover, exploring the underlying mechanisms of action and identifying response predictors can further refine these therapies’ use in clinical practice.

The results of the current review are consistent with previous findings that HA injections provide significant short-term relief of pain and improvement in joint function. For instance, Babu et al. [51] reported substantial improvements in VAS and KOOS scores with a single dose of HMW-IAHA. Similarly, a recent systematic review by Hunter et al. found that viscosupplementation with HA leads to significant pain reduction and improved function, albeit with some variability in outcomes due to differences in HA formulations and injection protocols. This aligns with the observation that HA’s efficacy can be influenced by molecular weight and additional components like corticosteroids [75].

In contrast, the effectiveness of PRP therapy has been highlighted in our review and other recent studies. Raeissadat et al. [52] and Nouri et al. [57] demonstrated that PRP, particularly when combined with HA, resulted in better long-term outcomes than HA alone. This is corroborated by a systematic review by Szwedowski et al. [58], which found that PRP injections led to significant improvements in pain and function, often outperforming HA and corticosteroids in long-term follow-ups. This superiority is attributed to PRP’s ability to promote tissue regeneration and reduce inflammation, providing more sustained relief than HA and corticosteroids’ temporary effects [76].

The variation in study designs, including differences in sample sizes, duration of follow-up, and the specific formulations or protocols used for PRP and HA, can significantly impact the outcomes. Some studies with more rigorous methodologies, such as randomized controlled trials with larger sample sizes and extended follow-up periods, show more reliable and consistent results. In contrast, studies with smaller samples or shorter follow-ups may produce less consistent findings, leading to apparent discrepancies in the results.

The discrepancies in the results between studies comparing PRP and HA highlight the complexity of treating knee osteoarthritis and the need for individualized treatment approaches. While some studies suggest PRP’s superiority, particularly in certain subgroups or under specific conditions, others do not find significant differences. These inconsistencies underscore the importance of considering the context of each study, including the methodology, patient population, and outcome measures, when interpreting the findings. Future research with more standardized protocols and well-defined patient cohorts must clarify these differences and provide more definitive guidance on using PRP versus HA in knee osteoarthritis. 

Additionally, the combination therapies involving corticosteroids and HA have shown mixed results. For instance, Hill et al. [56] reported that combining corticosteroids and HA provided significant short-term pain relief and physical function benefits. However, the long-term benefits were less clear, aligning with findings from a systematic review that indicated corticosteroids alone or combined with other agents offer limited long-term efficacy. This review also highlighted the potential for extended-release formulations of corticosteroids to provide prolonged joint residence time and better outcomes, an area currently under active research [77].

Following this review, it can be stated for clinicians that the combined administration of PRP and HA may be beneficial by improving pain and function.

The study by Lana et al. (2016) compared the synergistic use of HA and PRP for the treatment of mild and moderate osteoarthritis of the knee. They concluded that the combined use of the two products brought favorable results for developing functional capacity and alleviating pain [78].

Also, Zhao et al. showed that combined treatment of the two substances had an essential benefit for pain and function compared to PRP alone [79].

Research on combined PRP and HA therapy in knee OA has shown that patients with lower degrees of cartilage degeneration achieved superior results to those affected by advanced OA [80]. However, the ideal candidate for PRP and HA has never been reported [81].

Interestingly, the researchers found that combined therapy can also provide benefits to elderly patients with an advanced degree of osteoarthritis who are not eligible for prosthetics [82]. 

### 4.6. Strength and Limitations

Strengths of the Study:

Relevance to Current Practices: By exclusively incorporating recent studies, the review guarantees that its findings accurately represent the latest clinical practices, guidelines, and technologies, offering valuable insights for both clinicians and researchers to make well-informed decisions based on the most current evidence.

Advancements in Treatment Modalities: The review gains from the inclusion of studies integrating significant advancements in the formulation and delivery of hyaluronic acid (HA) products, providing a more precise assessment of the efficacy and safety of contemporary HA therapies, including the use of nanomaterials in intra-articular interventions [83].

Updated Methodological Standards: Recent studies are more likely to adhere to rigorous methodological standards, featuring improved randomization techniques, better control of confounding variables, and more sophisticated statistical analyses, thereby enhancing the reliability and validity of the findings [84]. 

Focus on Novel Interventions: The review encompasses studies exploring novel interventions and combination therapies, which are increasingly crucial in the evolving landscape of KOA treatment, offering insights into cutting-edge treatments not covered by older studies [85].

Minimizing Historical Bias: By concentrating on the last ten years, the review diminishes the influence of historical biases associated with outdated practices, less sophisticated diagnostic tools, and older HA formulations, ensuring a more accurate and relevant synthesis of current evidence [86].

Reflecting Current Epidemiology: Recent studies are more likely to reflect the current trends in KOA epidemiology and patient demographics, which have shifted due to changes in population health trends, lifestyle factors, and environmental influences, making the findings more applicable to today’s patient populations [87].

Despite the comprehensive nature of this systematic review, several limitations must be acknowledged:

Heterogeneity of Interventions: The studies included in this review examined a wide range of interventions, including different formulations and combinations of hyaluronic acid (HA), platelet-rich plasma (PRP), and corticosteroids. This heterogeneity makes direct comparisons challenging and may introduce variability in outcomes. For instance, different molecular weights of HA and various preparation methods for PRP can lead to different therapeutic effects, as highlighted by Blicharski et al. [53] and Raeissadat et al. [26,27,52].

Variability in Study Design: The inclusion of both RCTs and observational studies introduces potential biases and confounding factors. While RCTs are considered the gold standard, observational studies are susceptible to selection bias and lack randomization, which can affect the validity of the findings.

Inconsistent Follow-Up Periods: The follow-up periods varied significantly across studies, from a few months to several years. This inconsistency can impact the assessment of long-term efficacy and safety. Long-term studies, such as Galluccio et al. [61], provide valuable data on sustained outcomes, but shorter studies may not capture the full duration of treatment benefits [35].

Sample Size and Demographics: Some studies had small sample sizes, which can limit the generalizability of the results. Additionally, the demographic characteristics of participants varied, with some studies including a higher proportion of female participants or older adults. This variability can influence the outcomes and their applicability to the general population.

Publication Bias: There is a potential for publication bias, where studies with positive results are more likely to be published than those with negative or inconclusive outcomes. This bias can skew the overall assessment of treatment efficacy.

Exclusion of Long-Term Data: By excluding studies older than ten years, the review may overlook valuable long-term data that could provide insights into the sustained effects of intra-articular treatments over extended periods. Long-term outcomes are crucial in understanding the full impact of these therapies on KOA progression.

Potential Variability in Newer Studies: While recent studies follow updated methodological standards, there may be variability in how newer interventions are implemented and reported. This could affect the consistency and comparability of the results, mainly when novel therapies are involved. 

The review has the limitation of excluding non-English publications due to translation constraints, potentially introducing language bias and restricting the representation of global evidence on knee osteoarthritis therapies. It’s important for future reviews to consider including non-English studies with proper translation resources for a more comprehensive understanding of the topic. 

### 4.7. Contributions to the Scientific Domain and Clinical Practice

Despite these limitations, this systematic review makes significant contributions to both the scientific community and clinical practice:

Comprehensive Synthesis of Evidence: This review provides a thorough overview of the current state of intra-articular therapies for KOA by collating data from 23 diverse studies. It highlights the efficacy of various interventions, including HA, PRP, and combination therapies, thus informing future research directions.

Identification of Effective Interventions: The review underscores the effectiveness of HA and PRP in managing KOA, with several studies demonstrating significant improvements in pain and function. For example, Raeissadat et al. [52] and Nouri et al. [57] found that PRP, especially when combined with HA, often provides superior long-term outcomes compared to HA alone [26,31].

Insights into Combination Therapies: The review highlights the potential benefits of combination therapies, such as HA with PRP or corticosteroids. Studies like Nouri et al. [57] and Hill et al. [56] demonstrated that combining different therapeutic agents could offer enhanced outcomes, providing a rationale for further exploration of these synergistic effects [30,31].

Guidance for Clinical Practice: Clinicians can use this review’s findings to make informed decisions about KOA treatment options. The evidence supports using HA and PRP as effective interventions, with combination therapies offering additional benefits. This can help tailor treatment plans to individual patient needs, improving overall outcomes.

Highlighting Areas for Future Research: The review identifies gaps in the current literature, such as the need for standardized protocols and longer follow-up periods. Addressing these gaps in future studies can provide more definitive evidence on the long-term efficacy and safety of intra-articular therapies for KOA.

## 5. Conclusions

This thorough review emphasizes the potential of various intra-articular therapies in managing knee osteoarthritis (KOA). The wide range of study designs, comprehensive assessment methods, and diverse participant demographics provide a strong basis for understanding the effectiveness and safety of these treatments. While this review has its limitations, it significantly contributes to understanding intra-articular therapies for KOA. Synthesizing a broad range of studies offers valuable insights into effective treatments, guides clinical practice, and highlights areas for future research. This comprehensive analysis is a crucial resource for researchers and clinicians seeking to enhance the management of knee osteoarthritis. Future research should continue to explore combination therapies and long-term outcomes to optimize KOA management further.

## Figures and Tables

**Table 1:** Main characteristics of the analyzed studies.

NO.	Titles, Author, Year	Type of Study/Period	Participants	Type of Intervention	Evaluation Methods	Results
1	Babu et al., 2023 [51]	Prospective interventional cohort/March 2020–October 2022	44 participants, age-36–76 years, 73% women, 27% men. Average body mass index—25.85	HMW-IAHA The treatment involved a 90 mg dose of HMW-IAHA in 3 mL prefilled syringes.	VAS, KOOS, WORMS, Likert Scale	Over 90 days, the VAS score decreased from 8.53 to 5.97, indicating an improvement in pain levels. Additionally, the KOOS displayed both overall and specific enhancements during follow-up visits. Furthermore, the WORMS exhibited improvement, decreasing from 66.57 to 65.14 between day 0 and day 90.
2	Raeissad et al., 2021 [52]	Randomized clinical trial/December 2017–February 2019	200 participants 52 HA, 51 PRP, 49 PRGF, 48 Ozone Age 56.9 ± 6.3 Sex M/F-61/139	HA-Hyalgan (Fidia Farmaceutici S.P.A, Abano Terme, Italy) (3 doses per week), PRP—Royagen kit (made by Arya Mabna Tashkis Co., Tehran, Iran, SN: 312569) (two doses at three-week intervals), PRGF (two doses at three-week intervals), Ozone (Ozonibaric P ozone generator, by Sedecal, Madrid, Spain) (3 doses per week) IA injections were executed by a latero-median approach with the knee in extension.	VAS, WOMAC, Lequesne index	During the 2-month follow-up after the injection, lower values for WOMAC, Lequesne, and VAS scores were identified in the ozone group. At the 6-month evaluation, both the PRP and PRGF groups improved VAS and WOMAC scores compared with the HA group. When assessed at 12 months, the PRP and PRGF groups displayed significant differences compared to the HA and ozone groups.
3	Blicharski et al., 2023 [53]	Prospective randomized double-blind/January 2021–December 2021	284 participants aged ≥ 40 years, gender M/F-105/179	Hyruan ONE (LG Chem, Ltd., Seoul, South Korea), Durolane (Q-Med AB, Uppsala, Sweden)—intra-articular administration	WOMAC, Likert Scale, PGA, IGA, Kelgreen-Lawrence	The main outcome, measuring the mean change in WOMAC-Likert Pain sub-scores showed comparable improvements in both the test and comparator groups, with scores of −5.59 and −5.54, respectively, confirming the non-inferiority of the test product (difference of −0.05). The secondary outcomes, adverse events, and non-inferiority of Hyruan ONE to the comparator were consistent across both groups in European patients with mild-to-moderate knee osteoarthritis.
4	Calvet et al., 2022 [54]	Multicenter observational prospective with a single cohort	166 participants, age-63.2, sex M/F-40/126, BMI-28.6	Pronolis^®^ HD mono 2.5% (Procare Health, Spain/KD Intra-Articular® Gel 2.5%), the subjects were followed for 24 weeks.	Womac, Likert Scale, VAS	After a single injection of high-density HA viscoelastic gel, patients experienced a significant reduction in the WOMAC score by 4.78 points at the 12-week follow-up visit. This improvement resulted in noticeable relief from pain and better management of symptoms.
5	Acharya et al., 2022 [55]	Prospective observational/November 2021–January 2022	50 participants, sexM/F-21/29, age 45–55 years-27 patients Age-55–65 years-12 patients Age > 65 years-11 patients	One intra-articular injection of 10 mL of Hylan G-F 20 (Synvisc-One® (Hylan G-F 20)/(Hylan Polymer A & B G-F 20) Patients were followed per protocol at 8, 24, and 52 weeks.	WOMAC, VAS, SF 36, Kelgreen-Lawrence	A single intra-articular injection has been proven to significantly reduce pain intensity, enhance physical functioning, and elevate overall quality of life.
6	Hill et al., 2023 [56]	Multicenter, randomized, double-blind, placebo-controlled clinical trial/21 December 2018–25 October 2019	93 participants, age-30–75 years FP-MD-48, placebo-45	FlexPro MD^®^-(combination of Euphausia superba Antarctic krill oil (321 mg, Superba®, Aker BioMarine Antarctic US LLC.; Metuchen, NJ, USA), natural astaxanthin purified from Haematococcus pluvialis (2 mg, Zanthin® Natural Astaxanthin), and a proprietary HA produced from fermentation by Streptococcus zooepidemicus (30 mg, Flexonic® sodium hyaluronate (the sodium salt of HA), Valensa International; Eustis, FL, USA) (600 mg soft capsule consisting of krill oil, natural astaxanthin, and a proprietary HA). Therapy administration: 1 capsule/day, 12 weeks.	K-VAS, K-WOMAC, KSF-36, Kelgreen-Lawrence	In the FP-MD group, there was a reduction in K-VAS score from 46.1 to 25.3 at week 12. In the placebo group, the K-VAS score started at 42.7 and reached 32.1 at week 12. Regarding the K-Womac score, individuals receiving FP-MD experienced significantly lower levels of pain, stiffness, and improved physical function.
7	Nouri et al., 2020 [57]	Randomized clinical trial/6 April 2019–16 March 2020	92 participants: HA-29 participants, PRP- 32 participants, HA + PRP-31 participants. Gender M/F-25/67 Age: HA-60.93 ± 4.54 PRP-58.22 ± 5.10 HA + PRP-60.29 ± 4.83	In the HA group-2.5 mL intra-articular (Viscor 50 mg/2.5 mL, molecular weight of 2500–3200 kDa, Nitka, Tehran, Iran) In the PRP group-5 mL autologous PRP In the HA + PRP group, 5 mL of PRP was injected and immediately after that 2.5 mL of HA.	WOMAC, Lequesne index, VAS,	Based on the study findings, all three interventions effectively improved both pain and function, demonstrating significant differences in WOMAC, VAS, and Lequesne scores at 2 and 6 months compared to the baseline. These results highlight the positive impact of the interventions and underscore their potential to bring about meaningful improvements in patient outcomes.
8	Szwedowski et al., 2022 [58]	Prospective randomized/April 2019–March 2020	75 participants: PRP-25 participants, HA-25 participants, CS-25 participants Age of PRP-40–70 years HA-40–66 years CS-46–69 years.	Subjects were randomly assigned to receive intra-articular injections in one of three treatment groups: PRP (Density Platelet Gel, IBF, Scafati, Italy), HA (Biovisc Ortho Single, 30 mg/mL, molecular weight 3.400–3.800 kDa, Atradis Medical Devices, Warsaw, Poland), or CS (Diprophos, 6.43 mg/mL betamethasone dipropionate and 2.63 mg/mL betamethasone sodium phosphate, MSD, Warsaw, Poland), determined by a computer-generated randomization system.	Kelgreen-Lawrence score, WOMAC, BMI	The first stage involved evaluating the effectiveness of injections on the WOMAC scale at 1.5 months, 3 months, and 6 months. The PRP group demonstrated a more significant reduction in pain values compared to the HA group, although both groups experienced a decrease in pain. The administration of glucocorticoids resulted in the most significant decrease at 6 weeks.
9	Sconza et al., 2024 [59]	Prospective/November 2021	83 participants, sex M/F-37/46, age-47–87 years	Patients diagnosed with symptomatic KOA were administered a single intraarticular injection of HA-SC (SINOGEL®, IBSA Farmaceutici Italia srl, Lodi, Italy), a combination of 72 mg of sodium hyaluronate and 48 mg of sodium chondroitin,	VAS, WOMAC, Likert Scale, Kelgreen-Lawrence, PtGA	A WOMAC pain score reduced from point 7 to point 4 at 6 months post-treatment was reported. At 6 months post-administration, t he WOMAC score for physical function limitation decreased significantly from 26 to 13. Following a single IA injection of SINOGEL, the VAS pain score decreased notably from 6 to 4 at the 6-month mark post-injection.
10	Perruchet et al., 2023 [60]	Cross-sectional study/October 2021–February 2022	51 participants, gender M/F-18/33, age 66 ± 12 years, mean BMI-26.1	Patients were administered a singular 2.2 mL injection of HANOX-M-XL (HAPPYCROSS^®^; LABRHA SAS, Lyon, France), an extended-release viscosupplement that integrates cross-linking and mannitol. This unique formulation allows for a single-injection treatment, providing convenience and potential therapeutic benefits to the patients.	Kelgreen-Lawrence Score, radiological phenotype, BMI, DE (duration of effectiveness).	A single intra-articular injection of HANOX-M provides pain relief for approximately one year for patients with KL 1 and 2.
11	Galluccio et al., 2022 [61]	Cohort study/2015–2022	60 patients, sex M/F-29/31, average age 61.07 ± 9.15, average value of BMI-22.075 ± 2.42	Viscosupplementation with HA (HYALUBRIX—Fidia Farmaceutici S.P.A., Abano Terme, Italy)—A complete treatment course with one weekly injection of Hyalubrix for 3 consecutive weeks, followed by a single booster injection every 3 months until the end of the 5th year of follow-up.	Kelgreen-Lawrence score, WOMAC, NRS	Over the past five years, quarterly IA injections of hyaluronic acid (HA) have proven to be a safe and efficient treatment for alleviating pain and enhancing joint function.
12	Tschopp et al., 2023 [62]	Prospective single-center placebo-controlled study/February 2016–November 2019	95 patients, sex M/F-54/41, age-54–68 years	HA (sodium hyaluronate solution, “Suplasyn 1-shot”; Viatris, Canonsburg, PA, USA), TRIAMCINOLONE (triamcinolon, “Triamcort Depot”; Zentiva, Prague, Czech Republic), PRP On day 1 of the study, patients received 1 mL of Triamcinolone (Triamcort Depot) or 6 mL of HA (Suplasyn 1-Shot), or 3 mL of the subject’s PRP.	NRS, WOMAC, TAS	After receiving glucocorticoid treatment, the group experienced a significant reduction in pain, as confirmed by both NRS and WOMAC scores one-week post-injection. However, this effect disappeared after three months. The HA-treated group had minimal changes in NRS and WOMAC scores, with the most noticeable pain reduction occurring at 15 months. The results for the group treated with PRP were inconclusive.
13	Yılmaz et al., 2024 [63]	Retrospective/February 2020–February 2022	60 participants, sex M/F-13/47, mean age 57.9 ± 4.29 years	VS with cross-linked HA (2 mL) (SO Visc Cross-Linked; Biolot Medical, Ankara, Türkiye) compared with linear HA (2 mL) (SO Visc; Biolot Medical, Ankara, Türkiye)	WOMAC, OKS	Both injections exhibited a noteworthy enhancement from the baseline in WOMAC and OKS at 3 and 6 months.
14	Colombini et al., 2023 [64]	Single arm monomeric interventional/February 2021–April 2022	38 participants gender M/F-17/21, age 26–83 years	VS with CR500 gel (a peptide mixture in a 1.5 mL monodose vial. CR500® is formulated as follows: demineralized water, glycerin 99.8% PF, Propylene glycol, PEG-40 hydrogenated castor oil, preservative, carbomer, hyaluronic Acid HMW, xanthan gum, disodium EDTA, panthenol, sodium hydroxide, SH-Polypeptide-85 and SH-Polypeptide-93). The treatment was administered during the initial visit and then repeated at home by the patients on two consecutive days per week for 4 weeks.	KOOS, LKI	The total LKI score decreased statistically from baseline to final of the research. The analysis of the KOOS pain subscale definitively demonstrated a significant improvement in the patient’s condition at two, three, and four weeks compared to baseline.
15	Ciapini et al., 2023 [65]	Prospective/January 2018–January 2020	60 participants, age 39–80 years	Subjects were randomly divided into three groups, with 20 subjects in each group (10 males and 10 females). Group A received IAHA ArthroVisc (ArthroVisc; Regen Lab, Le Mont-sur-Lausanne, Switzerland), Group B received autologous intra-articular platelet-rich plasma (PRP) (RegenKit-BCT-1; Regen Lab, Le Mont-sur-Lausanne, Switzerland), and Group C received an association of substances of plasma and HA (Cellular Matrix A-CP-HA kit; Regen Lab, Le Mont-sur-Lausanne, Switzerland). Each group underwent three intra-articular injections over the course of 2 months.	WOMAC, VAS	In Group A, the average VAS score started at 5.5 and lowered to 4.3 after 3 months, stabilizing at 4 after 6 months. The WOMAC score was 36.4 initially, decreased to 28.8 at 3 months, and increased to 31.8 at 6 months. In Group B, the mean VAS score was 6.1 before injections, decreased to 3.1 after 3 months, and remained at 3.5 after 6 months. The WOMAC score was 41.5 initially, decreased to 19.6 after 3 months, and remained unchanged in the subsequent 3 months.
16	Tammachote et al., 2016 [66]	Participants were recruited in a prospective, randomized, double-blind clinical trial. The treatment group was kept secret from patients and evaluators.	110 participants with knee osteoarthritis (KOA) were randomly assigned to receive either hylan G-F 20 or a triamcinolone acetonide injection.	1. Administer a single IA injection of 6 mL of hylan G-F 20 (Synvisc; Genzyme Biosurgery, Cambridge, MA, USA), a viscosupplement used to relieve joint pain. 2. Administer a single IA injection of 1 mL of 40-mg triamcinolone acetonide combined with 5 mL of 1% lidocaine hydrochloride and epinephrine for anti-inflammatory and analgesic effects.	Knee pain-100-mm VAS, WOMAC	After six months, triamcinolone acetonide exhibited equivalent improvements in knee pain, function, and range of motion compared to Hylan G-F 20. Furthermore, in contrast to Hylan G-F 20, triamcinolone acetonide exhibited superior pain control during the initial week and enhanced knee function during the subsequent week.
17	Hermans et al., 2019 [67]	RCT of subjects aged 18–65 with symptomatic KOA (Kellgren-Lawrence grade I–III)	Subjects were assigned at random to either receive standard care (control group) or standard care along with three weekly injections of high molecular weight hyaluronic acid (intervention group).	The study intervention involved three weekly injections of high-molecular-weight hyaluronic acid (Hylan G-F 20—Sanofi S. A, Paris, France), additionally to conventional care, including pain medication, physical therapy, and lifestyle recommendations.	OMERACT-OARSI criteria KOOS, NRS, Likert scale	High molecular weight hyaluronic acid (HMW-HA) injections led to a superior response to therapy and substantial improvements in pain relief, knee function, and overall assessment compared to the control group during the 52-week follow-up period. While the intervention group initially experienced temporary knee pain and swelling in the first 6 weeks, no serious adverse events were reported.
18	Bashaireh et al., 2015 [68]	Prospective, nonrandomized, unblinded, phase IV, multicenter, post-marketing study design	109 participants enrolled, with 84 completing all visits	The intervention in this study was a single intra-articular injection of 2 mL of Crespine^®^ Gel (Biopolymer GmbH & Co. KG, Dümmer, Germany), a cross-linked hyaluronic acid product.	QoL WOMAC	The use of Crespine^®^ Gel, a cross-linked hyaluronic acid product, unequivocally improved pain, stiffness, and physical function in subjects with KOA. The effects lasted for up to nine months following a single injection. Furthermore, the treatment demonstrated excellent tolerability, with predominantly mild and temporary local adverse events.
19	Ertürk et al., 2016 [69]	Single-blinded, randomized, prospective controlled trial	Two groups of patients were formed. One group received hyaluronic acid injections directly into the joint, while the other received a combination of hyaluronic acid injections into the joint and a single corticosteroid-lidocaine injection around the joint.	The treatment groups received either five weekly 2.5 mL injections of 900,000 Da sodium hyaluronate (10 mg/mL, Adant®, Meiji Seika Kaisha Co, Tokyo, Japan). Ethyl chloride spray (IGS AEROSOLS GMBH, D-79664 Wehr/Baden, Germany)) directly into the knee joint (IA), or a single injection of 1 mL of betamethasone dipropionate (6.43 mg) and betamethasone sodium phosphate (2.63 mg) mixed with 1 mL of 20 mg lidocaine (Diprospan®; Schering-Plough, Istanbul, Turkey) mixed in 1 ml:20 mg of lidocaine HCl without epinephrine (Jetokain simplex®; Adeka, Istanbul, Turkey) HCl into the most painful areas of the knee, in addition to the hyaluronic acid injections (periarticular lidocaine-corticosteroid injection).	VAS pain scale, WOMAC, and HSS knee scores.	Adding a periarticular lidocaine-corticosteroid injection (PALCI) to intraarticular hyaluronic acid (HA) treatment provided better pain and functional outcomes in the first 3 weeks compared to HA alone, but the differences were not significant after 6 weeks. The combined PALCI and HA treatment can offer earlier pain relief and help patients return to daily activities sooner compared to HA alone. Some minor adverse events were observed with the combined treatment, but no serious adverse events were reported.
20	Rici et al., 2017 [70]	clinical comparison study.	A total of sixty patients, comprising 32 males and 28 females aged between 40 and 70 years, were methodically assigned to two separate research study groups.	The study involved two treatment groups. Group A received three weekly injections of 1.6% hyaluronic acid directly into the joint, while Group B took Syalox (River Pharma, Orio Litta, Italy) 300 Plus (which contains 300 mg hyaluronic acid and 100 mg Boswellia serrata extract) orally for 20 days, followed by Syalox 150 (containing 150 mg hyaluronic acid) for another 20 days. Both treatments showed positive effects on individuals with early osteoarthritis. The results indicated that using both treatments together could be beneficial, especially for different age groups.	AKS, VAS	The research paper found that both hyaluronic acid (HA) injections and oral administration positively affect early osteoarthritis patients. The treatment led to significant improvements in AKSS and VAS scores. It was observed that younger patients experienced greater benefits from injections, while older patients showed improved outcomes with oral administration. These findings strongly suggest that a combined therapy approach could be highly effective. The study also highlights the potential of oral HA absorption and distribution to joints.
21	Tang et al., 2015 [71]	Observational, comparative clinical trial	The study included 23 subjects with KOA and 14 age-matching control subjects without knee osteoarthritis from an outpatient clinic	Bilateral intra-articular knee joint injections with hyaluronic acid (HA) were administered to the knee OA group using a 1% HA solution (ARTZ). The injections were administered at 2.5 mL per joint/weekly for 5 consecutive weeks.	Muscle co-contraction and motor response of quadriceps, hamstrings, tibialis anterior, and medial gastrocnemius	The paper’s research shows that injecting hyaluronic acid into the knee joint can change muscle activation patterns. In a study involving 23 knee osteoarthritis patients and 14 control subjects, the injections improved muscle activation, reduced co-contraction, and enhanced motor activity. These improvements lasted for up to six months after treatment, demonstrating the effectiveness of hyaluronic acid in altering muscle activation patterns in knee osteoarthritis patients.
22	Lisi et al., 2018 [72]	Phase-2 randomized controlled trial.	The study comprised 156 participants, with 77 in the intervention group and 79 in the control group. All participants exhibited symptomatic KOA and were aged between 18 and 65	In the intervention group, patients underwent a series of three autologous PRP alongside calcium gluconate injections, whereas the control group received three hyaluronic acid injections (20 mg/2 mL; Hyalgan; Fidia, Abano Terme, Italy).	MRI scans and functional scales like WOMAC, Lysholm, Tegner, AKSS, Lequesne, and VAS for pain	The MRI scans showed improvement in twenty-eight patients in the intervention group and twenty-two in the control group six months after treatment. It is evident that activated platelet-rich plasma effectively reduced joint damage and improved pain, function, and quality of life for at least one year. The treatment group consistently exhibited superior improvements in symptoms and functional scales compared to the control group, and these improvements were statistically significant across various scales.
23	Di Martino et al., 2019 [73]	Randomized Controlled Trial-5 years follow-up	The population sample size for the study was 192 patients who were enrolled in RTC comparing PRP and HA administrating for KOA	One group of 85 participants received three weekly intra-articular injections of leukocyte-rich PRP, while the other group of 82 participants received three weekly intra-articular injections of high-molecular-weight HA (Hyalubrix 30 mg/2 mL, molecular weight > 1500 kDa, Fidia Farmaceutici S.P.A., Abano Terme, Italy).	International Knee Documentation Committee (IKDC) subjective score, EuroQol VAS, Tegner score.	Both PRP and HA therapies demonstrated effectiveness in improving knee function and symptoms. At the final assessment, PRP exhibited higher values compared to baseline; however, It did not demonstrate a significantly better clinical improvement compared to HA. The PRP group exhibited a notably lower rate of reintervention at 24 months.

KOOS—Knee Injury and Osteoarthritis Outcomes Score, VAS—Visual analog Scale, K-VAS—Korean Visual analog Scale, WORMS—Whole Organ Magnetic Resonance Imaging, OKS—Oxford Knee Score, WOMAC—The Western Ontario and McMaster Universities Arthritis Index, K-WOMAC—Korean Western Ontario and McMaster Universities Arthritis Index, KSF-36—Korean Medical Outcome Study 36-Item Short Form, PGA—Patient Global Assessment, IGA—Investigator Global Assessment, HAQ—Health Assessment Questionnaire, SF 36—Short Form 36 Health Survey, ROS—Reactive Oxigen Species, MW—Molecular Weight, LMW—Law Molecular Weight, HMW—Height Molecular Weight, BMI—Body Mass Index, PAGA—Patient Global Assessment of disease activity, NRS—Numeric Rating Pain Scale, IAHA—Intraarticular Hyaluronic Acid, HMW-IAHA—High Molecular Weight-Intraarticular Hyaluronic Acid, PRP—Platelet Rich Plasma, PRGF—Plasma Rich in Growth Factor, VS—Viscosupplementation, HA-SC—Hyaluronic Acid Sinogel, TAS—Tegner Activity Scale, LKI—Lequesne Knee Index.

## Supplementary Materials

The following are available online at https://www.mdpi.com/article/10.3390/clinpract14050157/s1, Supplementary Materials: Detailed Search Strategy.
