# Supplementary material for: The Potential of Intra-Articular Therapies in Managing Knee Osteoarthritis: A Systematic Review
Source: Clin Pract. 2024 Sep 25;14(5):1970–96. doi: 10.3390/clinpract14050157 (PMC11506573; doi:10.3390/clinpract14050157)
Supplement: Supplementary file 1 [file clinpract-14-00157-s001.zip › clinpract-3178806-supplementary.pdf]

Supplementary Materials: Detailed Search Strategy

1. Database Selection

The search was conducted across three major electronic databases to ensure comprehensive coverage of the literature:

1. **MEDLINE (via PubMed)**
2. **Web of Science Core Collection**
3. **ScienceDirect Collection (Elsevier)**

These databases were chosen for their extensive collection of biomedical and clinical research literature, ensuring that relevant studies on intra-articular therapies for knee osteoarthritis (KOA) were captured.

2. Search Terms and Query Construction

A combination of specific keywords and Medical Subject Headings (MeSH) terms was used to capture relevant studies. The search terms were selected based on their relevance to knee osteoarthritis and the interventions of interest (hyaluronic acid and platelet-rich plasma).

Primary Search Terms:

- "Knee"
- "Osteoarthritis"
- "Hyaluronic acid"
- "Viscosupplementation"
- "Intra-articular injection"
- "Platelet-rich plasma"
- "PRP"

MeSH Terms:

- "Osteoarthritis, Knee"
- "Hyaluronic Acid"
- "Injections, Intra-Articular"
- "Platelet-Rich Plasma"

Boolean Operators:

- The terms were combined using Boolean operators (AND, OR) to refine and broaden the search as needed.

("Knee"[MeSH] OR "Knee") AND ("Osteoarthritis"[MeSH] OR "Osteoarthritis") AND ("Hyaluronic Acid"[MeSH] OR "Hyaluronic acid" OR "Viscosupplementation") AND ("Injections, Intra-Articular"[MeSH] OR "Intra-articular injection") AND ("Platelet-Rich Plasma"[MeSH] OR "Platelet-rich plasma" OR "PRP")

Additional Filters:

- **Language:** English
- **Publication Date:** Studies published in the last 10 years (2014-2024)
- **Study Type:** Randomized Controlled Trials (RCTs), cohort studies, and prospective observational studies

3. Search Execution

The search was conducted from March 4th to March 8th, 2024. Each database was queried separately using the constructed search terms. The results from each database were exported into a reference management software (Zotero) for further processing.

4. Screening and Selection Process

1. **Duplicate Removal:**
    - All retrieved articles were imported into Zotero. Duplicates were identified and removed automatically by the software to avoid redundant data.
2. **Title and Abstract Screening:**
    - The titles and abstracts of the remaining articles were screened for relevance. Articles that did not meet the inclusion criteria were excluded at this stage.
3. **Full-Text Review:**
    - The full texts of potentially relevant articles were obtained and reviewed in detail. Studies were included if they met the predefined inclusion criteria.

5. Inclusion and Exclusion Criteria

Inclusion Criteria:

1. Studies involving patients diagnosed with knee osteoarthritis.

2. Studies evaluating the efficacy of hyaluronic acid (HA) or platelet-rich plasma (PRP) for intra-articular injections.
3. Studies reporting on pain, function, or overall treatment efficacy outcomes.
4. Studies published in English and within the last 10 years.
5. Both RCTs and observational studies were included.

Exclusion Criteria:

1. Non-original research articles (e.g., reviews, commentaries, editorials).
2. Animal studies or in vitro experiments.
3. Studies published in languages other than English.
4. Unpublished data (e.g., conference abstracts, dissertations).
5. Studies with incomplete data or insufficient methodology reporting.
6. Research focused on interventions other than intra-articular HA or PRP (e.g., oral medications, surgical interventions).
7. Studies with a follow-up period of less than 3 months.
8. Studies with fewer than 20 participants per treatment group.
9. Duplicate publications of the same study, where the most comprehensive version was included.

6. Data Extraction and Analysis

For each included study, the following data were extracted:

- Study design (e.g., RCT, cohort study)
- Sample size
- Participant demographics (age, gender distribution)
- Duration of follow-up
- Types of interventions (specific HA formulations, combinations with other treatments)
- Outcome measures (e.g., VAS for pain, WOMAC for function)
- Key findings and results

7. Quality Assessment

The quality of the included studies was assessed using the Cochrane Collaboration's risk of bias tool, which evaluated potential biases such as selection, performance, detection, attrition, and reporting biases.

Screening and Review Process

The screening and review of the articles were a collaborative effort involving multiple authors to ensure a thorough and unbiased selection of studies:

- **Initial Screening:** C.V.P. (Ciprian Vasile Pojala) and C.E.P. (Cristiana Elena Pojală) conducted the initial screening of titles and abstracts under the supervision of **L.D. (Lorena Dima)**. They reviewed the abstracts to assess the relevance of the studies according to the predefined inclusion criteria.
- **Full-Text Review:** The full-text articles that passed the initial screening were reviewed in detail by **N.A.R. C.V.P.** and **C.E.P. N.A.R.** led the methodological assessment during this stage to ensure consistency in applying the inclusion and exclusion criteria.
- **Validation:** The selected studies were then validated by **L.D., and C.V.P.** and **S.T.** to confirm their relevance and inclusion in the systematic review. This validation process involved cross-checking the studies to ensure no critical articles were missed and verifying the accuracy of the data extracted.

Bias Analysis

The bias analysis was an integral part of the systematic review to assess the quality of the included studies:

- **Methodology and Bias Analysis: N.A.R.** was primarily responsible for the bias analysis. She applied the Cochrane Collaboration's risk of bias tool to assess selection, performance, detection, attrition, and reporting biases in the included studies.
- **Formal Analysis:** The formal analysis, which included interpreting the bias assessment and its impact on the study outcomes, was conducted by **C.C. and C. E. P.** provided a detailed analysis of how potential biases could affect the reliability of the study findings.
- **Supervision:** The bias analysis was supervised by **L.D. and N.A.R.** who ensured that the process was rigorous and that the conclusions drawn were well-supported by the data.

Step 1: Title Screening

Objective: To quickly identify and exclude studies that are clearly irrelevant to the research question based on their titles.

Process:

1. **Compilation of Search Results:**
   - All articles retrieved from the database searches were compiled into a reference management software (e.g., Zotero).
2. **Initial Title Screening:**
   - **C.V.P.** and **C.E.P.** independently screened the titles of all retrieved articles.
   - Titles that clearly did not match the inclusion criteria (e.g., studies focusing on unrelated medical conditions or non-human studies) were excluded at this stage.
3. **Discussion and Consensus:**
   - **C.V.P.** and **C.E.P.** compared their lists of excluded titles and discussed any discrepancies.
   - In cases where there was uncertainty about whether to exclude a title, the article was retained for abstract screening.
4. **Validation:**
   - The initial list of included titles was validated by **L.D.** to ensure no relevant studies were prematurely excluded.

Step 2: Abstract Screening

Objective: To assess the relevance of studies based on their abstracts and to further narrow down the list of potentially eligible studies.

Process:

1. **Abstract Review:**
   - **C.V.P.** and **C.E.P.** independently reviewed the abstracts of the studies that passed the title screening.
   - Abstracts were assessed against the inclusion criteria, focusing on key elements such as study population, interventions, outcomes, and study design.
2. **Inclusion/Exclusion Decision:**
   - Studies that clearly met the inclusion criteria were marked for full-text review.
   - Studies that did not meet the criteria (e.g., reviews, case reports, animal studies) were excluded.
   - Studies where the abstract did not provide sufficient information to make a decision were retained for full-text review.
3. **Consensus Meeting:**

- C.V.P. and C.E.P. discussed the abstracts they had reviewed, resolving any disagreements by consensus.
  - **N.A.R.** provided additional input on methodological aspects, ensuring consistency in the application of criteria.
4. **Validation:**
   - **L.D.** reviewed the final list of abstracts selected for full-text review to confirm the accuracy and appropriateness of the inclusion decisions.

Step 3: Full-Text Review

Objective: To conduct a thorough evaluation of the full-text articles to confirm their eligibility for inclusion in the systematic review.

Process:

1. **Full-Text Retrieval:**
   - Full-text articles were obtained for all studies that passed the abstract screening stage.
   - **C.V.P.**, **C.E.P.**, and **N.A.R.** were responsible for obtaining and organizing the full texts.
2. **Detailed Review:**
   - **N.A.R.** led the detailed full-text review, examining each article for alignment with the inclusion criteria, including study design, participant characteristics, interventions, outcomes, and the quality of the study.
   - Key aspects such as sample size, duration of follow-up, types of interventions, and outcome measures were carefully evaluated.
   - **C.V.P.** and **C.E.P.** assisted in the review process, particularly focusing on clinical relevance and the applicability of findings.
3. **Data Extraction:**
   - Data were extracted from eligible studies, including details about study design, participant demographics, interventions, and outcomes.
   - The extracted data were organized into tables for easier comparison and synthesis.
4. **Final Inclusion Decision:**
   - Studies that fully met the inclusion criteria were included in the systematic review.
   - Studies that did not meet the criteria, had methodological flaws, or lacked sufficient data were excluded.
   - The reasons for exclusion at this stage were documented.
5. **Consensus and Validation:**

- **L.D.** and **S.T.** validated the final list of included studies, ensuring that the selection was rigorous and aligned with the review's objectives.
- Any discrepancies or uncertainties during the full-text review were resolved through group discussion, with **N.A.R.** ensuring methodological soundness.

Step 4: Bias Analysis

Objective: To assess the risk of bias in the included studies, ensuring that the findings are reliable and valid.

Process:

1. **Methodological Assessment:**
    - **N.A.R.** conducted a thorough bias analysis using the Cochrane Collaboration's risk of bias tool.
    - Key biases, including selection bias, performance bias, detection bias, attrition bias, and reporting bias, were assessed for each study.
2. **Formal Analysis:**
    - **C.C.** performed the formal analysis of the bias assessments, identifying potential impacts on the study outcomes and overall reliability.
    - **C.V.P.** and **C.C.** worked together to interpret the results of the bias analysis.
3. **Supervision and Validation:**
    - **L.D.** and **N.A.R**. supervised the entire bias analysis process, ensuring that the assessments were thorough and consistent with best practices.
4. **Documentation:**
    - The results of the bias analysis were documented and included in the review to provide transparency and context for interpreting the findings.
